# Uniqueness of RNA Coliphage Qβ Display System in Directed Evolutionary Biotechnology

**DOI:** 10.3390/v13040568

**Published:** 2021-03-27

**Authors:** Godwin W. Nchinda, Nadia Al-Atoom, Mamie T. Coats, Jacqueline M. Cameron, Alain B. Waffo

**Affiliations:** 1 Laboratory of Vaccinology and Biobanking, International Reference Centre CIRCB), BP 3077 Yaoundé, Cameroon; nsehleseh@gmail.com; 2 Department of Pharmaceutical Microbiology & Biotechnology, Nnamdi Azikiwe University, 420110 Awka, Nigeria; 3 Department of Pathobiology, College of Veterinary Medicine, Tuskegee University, Tuskegee, AL 36088, USA; nal-atoom7321@tuskegee.edu; 4 Clinical and Diagnostic Sciences, University of Alabama at Birmingham, Birmingham, AL 35294, USA; mamiec@uab.edu; 5 Biochemistry and Molecular Biology, Indiana University School of Medicine, Indianapolis, IN 46202, USA; jacmcame@iu.edu

**Keywords:** M13, pIII, Qβ, *Leviviridae*, A_1_, A_2_, replicase, UGA, tryptophan, icosahedron, evolutionary biotechnology, in vitro evolution, affinity maturation, F^+^, minor coat, major coat, fitness landscape, quasispecies

## Abstract

Phage display technology involves the surface genetic engineering of phages to expose desirable proteins or peptides whose gene sequences are packaged within phage genomes, thereby rendering direct linkage between genotype with phenotype feasible. This has resulted in phage display systems becoming invaluable components of directed evolutionary biotechnology. The M13 is a DNA phage display system which dominates this technology and usually involves selected proteins or peptides being displayed through surface engineering of its minor coat proteins. The displayed protein or peptide’s functionality is often highly reduced due to harsh treatment of M13 variants. Recently, we developed a novel phage display system using the coliphage Qβ as a nano-biotechnology platform. The coliphage Qβ is an RNA phage belonging to the family of *Leviviridae,* a long investigated virus. Qβ phages exist as a quasispecies and possess features making them comparatively more suitable and unique for directed evolutionary biotechnology. As a quasispecies, Qβ benefits from the promiscuity of its RNA dependent RNA polymerase replicase, which lacks proofreading activity, and thereby permits rapid variant generation, mutation, and adaptation. The minor coat protein of Qβ is the readthrough protein, A_1_. It shares the same initiation codon with the major coat protein and is produced each time the ribosome translates the UGA stop codon of the major coat protein with the of misincorporation of tryptophan. This misincorporation occurs at a low level (1/15). Per convention and definition, A_1_ is the target for display technology, as this minor coat protein does not play a role in initiating the life cycle of Qβ phage like the pIII of M13. The maturation protein A_2_ of Qβ initiates the life cycle by binding to the pilus of the F^+^ host bacteria. The extension of the A_1_ protein with a foreign peptide probe recognizes and binds to the target freely, while the A_2_ initiates the infection. This avoids any disturbance of the complex and the necessity for acidic elution and neutralization prior to infection. The combined use of both the A_1_ and A_2_ proteins of Qβ in this display system allows for novel bio-panning, in vitro maturation, and evolution. Additionally, methods for large library size construction have been improved with our directed evolutionary phage display system. This novel phage display technology allows 12 copies of a specific desired peptide to be displayed on the exterior surface of Qβ in uniform distribution at the corners of the phage icosahedron. Through the recently optimized subtractive bio-panning strategy, fusion probes containing up to 80 amino acids altogether with linkers, can be displayed for target selection. Thus, combined uniqueness of its genome, structure, and proteins make the Qβ phage a desirable suitable innovation applicable in affinity maturation and directed evolutionary biotechnology. The evolutionary adaptability of the Qβ phage display strategy is still in its infancy. However, it has the potential to evolve functional domains of the desirable proteins, glycoproteins, and lipoproteins, rendering them superior to their natural counterparts.

## 1. Introduction

Phage display and engineering are powerful tools in synthetic biology and directed evolutionary biotechnology [1,2,3,4,5]. A phage represents a unique, yet miniature, simple model system that is easy and safe to manipulate [6,7,8]. Structurally, the minor and major coat proteins of phages can be genetically modified to encapsulate foreign peptides and present them upon their surface [9,10]. The modified phage genome contains the gene of the surface exposed foreign peptides. The linkage between the modified phage genome and the surface exposed foreign peptide is a unique and powerful tool for directed evolutionary study [11,12]. Through combinatorial cDNA libraries, room is created for more genetic insertions, with the possibility of expressing and exposing a further expanded novel phenotype. The diversity of a library in in vitro directed evolution is of great importance, as it increases the number of probes exposed, as well as diversity in target(s) directed specificity. Like viruses, phages may contain a DNA genome, which replicates using DNA-dependent-DNA-polymerase (DdDp) or an RNA genome, which uses an RNA-dependent-RNA-polymerase (RdRp) [13,14,15,16]. The RdRp and DdDp function differently and can affect the diversity of the library. The DdDp is known to contain a proofreading activity, which can stabilize or significantly reduce the diversity of a library [17,18]. However, the RdRp lacks proofreading activity, which in effect drives library diversity through error prone phage replication, and promoting a rapid evolution [19,20].

Bacteriophages were discovered independently by Felix d’Herelle and Frederick Twort in the 19th century, and have since been intensively studied [21,22,23,24]. Phage classification and genetics are well understood, but only M13 has been dominantly used in display and surface engineering [1,2,3,4,5,25,26,27,28]. Given the broad range of diverse phage structure and organization, it seems likely that directed evolution is applicable with other type of phages [29]. However, M13, the first phage used for foreign peptide display, remains dominant in the phage display field and engineering biotechnology [1,2,3,4,5,6,7,8,9,10]. M13 is cylindrical in shape, being 1 um in length, less than 10 nm in diameter and belongs to the group of phages referred to as Inoviridae, bearing a single stranded circular DNA core [30,31,32,33]. The M13 phage genome is about 6.4 kb, encoding for five different proteins, including p3, p6, p7, p8, and p9. Thus far, DNA phage M13 and phagemids have been commonly used despite tremendous advances in phage research [34,35,36,37]. The phage M13 minor protein p3 or pIII is commonly used to expose foreign peptides [36]. The protein pIll functionally directs virion tropism and infection by recognizing and binding to the pilus of F^+^ bacteria host. The dual functions of pIII in the phage life cycle limits its applicability in phage display biotechnology by reducing its efficiency during panning and amplification on target [1,2,3,4,5,6,7,8,9,10,36]. Additionally, the infectivity of the recombinant phages can be drastically reduced when pIII displayed foreign peptide probe are bound to their respective target. This necessitates harsh treatment, including heat or acidic elution and neutralization of the complex probe-target for M13 [1,8]. The phage Qβ belongs to Leviviridae family, with a small positively stranded RNA virus, with a 4.2 kb genome encoding 4 proteins including the coat (Cp), maturation (A_2_), readthrough (A_1_), and replicase (RdRp) protein [38]. As an RNA virus, the Qβ phage possesses the replicase protein, a key feature making it suitable for rapid evolution and adaptation [39]. The Qβ phage replicase is devoid of proofreading activity during replication, resulting in heightened mutation rates, simulating the affinity-maturation processes, promoting directed evolutionary biotechnology [19,20,39,40,41,42].

This review addresses the uniqueness of the novel RNA coliphage Qβ display system, specifically as an alternative to the traditional M13 display technology. Recently, this alternative system has shown progress in its application for immunogen display and monitoring of immunity of infectious diseases at point of care. We will discuss the unique capability of Qβ in library display, developed bio-panning for screening and hybrid phage amplification. We will also stress some major applications of the Qβ display system, such as presenting various types of surface proteins as immune check point inhibitors, antiviral therapy, and in the development of point of care nanobiotechnology using directed evolution.

## 2. RNA Evolution

### 2.1. Overall Evolution

Referring to Darwin, evolution was defined as the fitness of any living entity (wherever) which can survive and replicate under the present conditions. It is the ability to change (phenotype), adapt, and to replicate the genetic materials (genotype) through natural pressure to achieve the so-called “natural selection” [43,44]. This fitness went from a simple single concept to a complex fitness landscape, representing the quantitative living system [45,46,47]. As defined, the landscape is directly proportional to the genomic size. Therefore, the fitness landscape is complex for eukaryotic systems and limited physically by the huge number of different methods and sequences. Viruses represent a powerful tool to assess the fitness landscape, particularly phages. Bacteriophages represent a safe dynamic tool that would continue to drive and empower this concept. Phages are perfect natural biological tools for directed evolution studies. The power of the fitness landscape does not rely only on the genotype, but on the ability of the replicative machinery to do the replication process (DNA-based or RNA-based). The development of phage display technology has expanded the fitness landscape concept to include the ability to prepare, expose, use for screening different size, and format libraries of peptides on the phages. This is the insertion of synthetic biology in the field of biotechnology and in vitro evolution (IVE) into the era of evolutionary biotechnology, specifically the identification of desired functionalities from a single library of variants. IVE is efficient process, especially when considering RNA-based replication systems and their associated suitable features. Additionally, the coliphage Qβ, like other RNA viruses, has the unique position of belonging to the fitness landscape and performing as a rapid IVE tool.

### 2.2. Evolution with RNA Viruses

RNA viruses with their small genomes represent a true model of rapid evolution, yielding a large population size with inconsistency in the fitness landscape and short replication time [48]. The RNA virus population is often named the “quasispecies”, referring to the fact that the genomics of the population consist of a mutant spectrum rather than a single species with identical nucleotide sequence [49,50,51,52,53]. The existence of RNA viruses as a mutant spectra is a direct consequence of evolution. Many studies have shown the high rate of nucleotide misincorporation during replication from the parental template to the progeny viral genomes. The quasispecies can have advantages and disadvantages for the host (human) and the virus. An accumulation of mutations can lead to extinction (death) of the virus or other biological implications, like changing the cell tropism or host range and overcoming selective constraints. These selective constraints include antiviral drugs and immune therapy molecules (specific antibodies). The quasispecies existence of RNA viruses make it very difficult to detect and challenging to prevent and treat. Mutant swarms of virus specific targets can compromise the detection, especially at point of care.

There is a need to conceptualize and develop models of detection, treatments or prevention that will consider the viral quasispecies evolution. The uniqueness of the Qβ phage rapid evolution can fulfill this requirement and its adaptive potential can bridge the knowledge gap in combating the mutant clouds of infectious RNA viruses. The phage Qβ would not be an exception to the rules of RNA viruses, but would be a powerful tool for an IVE study instead. In fact, RNA Qβ phage could be used as a toolkit that would employ and exploit its unique features in the directed evolutionary process [54,55,56,57,58].

### 2.3. An Emphasis of Evolution with Coliphage Qβ

The Qβ phage possesses the major features of viral quasispecies. Specifically, great genetic variation due to the promiscuity of its RdRp, which lacks proofreading activity; competition and selection ability; viral fitness and effect of the population size; bottleneck events; fitness gain and change [47,48,49,50,51,52,53,54,55]. Genetic variation was observed with accumulation of point mutations in foot and mouth disease virus (FMDV), an evolutionary study with few internal genomic deletions that were severely infectious and killed cells between several passages (143 to 260) [47,50,52,53]. Exhibiting genetic variation in order to gain more function in the life cycle is ideal for the Qβ phage, as it is necessary to evolve and adapt to the new target during panning. Recently, we described a benchtop in vitro evolution system with RNA Qβ displaying a randomized VP1 GH loop peptide of FMDV [55]. The gene fusion of A_1_ and GH loop was stable in a replicating component that yields hybrid phages. The Qβ displaying on its surface localized the FMDV VP1 GH loop and cross-reacted with an anti-FMDV monoclonal antibody (mAb) SD6 and was found to decorate the 12 corners of the phage icosahedron. Hybrid phages presenting the GH loop selected under evolutionary pressure revealed a non-canonical (Arg-Gly), but essential epitope, for the mAb SD6 recognition motif [55]. Additionally, the competition amongst mutants for the target will agree with the Red Queen hypothesis, that states that occasionally superior mutants arise and exclude others by competition. Recently, foreign peptide genes, up to 80 mer, have been stably inserted into the phage genome and exposed to its surface. A library format with this size can be inserted into the phage as well [56,57]. In combination with innovative emergent technology, an intelligent library design avoiding inactive variants can be developed. Designing active variants within a library will give additional advantages to RNA virus evolution and strengthen IVE. The complexity of the mutant spectrum and the RNA phage display can act as a combination therapy and possibly ideal against RNA virus infections. The Qβ phage is unique, thus proving to be a major candidate, as well as perfect tool for directed evolution and IVE.

## 3. Uniqueness of the Biology Coliphage Qβ

**Figure 1:**
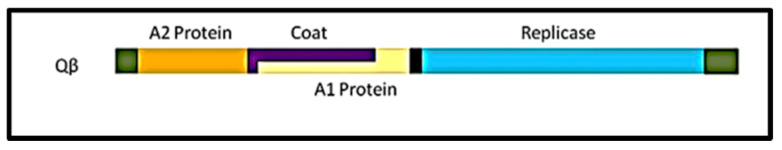
Genome organization of the RNA coliphage Qβ. A_2_ protein gene or maturation gene (from 62–1321 bases); Cp or major coat protein gene (from 1345–1743 bases); A_1_ protein gene or readthrough (from 1345–2331 bases); Replicase or the β-subunit gene of the RNA-dependent-RNA-polymerase (from 2353–4119 bases). The 5′ and 3” ends are flanked by non coding bases like any other open reading frame on this genome.

The Qβ coliphage belongs to the family of Leviviridae and exhibits a positive-sense single stranded RNA genome molecule (+ssRNA) [59]. Morphologically, Qβ particles are icosahedral in shape with 25 nm in diameter and a T = 3 triangulation [60]. Qβ is very simple with 4.1 kilobases genome (Figure 1) encoding for only four proteins including the maturation protein (A_2_), the coat protein (Cp), the minor structural protein (A_1_), and the replicase subunit (Rp) [61].

### 3.1. Coliphage Qβ Life Cycle

#### 3.1.1. Overall Cycle

Qβ phage infects various bacterial hosts with pilus structures. The so-called appendage pilus is commonly used for conjugation and plasmid transfer in those bacteria [62]. Physiologically, the F-pili functions to bring together the donor and recipient bacteria for the purpose of transferring the appropriate genetic material [63]. After attachment to the pili on the host cell surface, the Qβ phage particles eject the RNA-A_2,_ allowing the RNA to repurpose the gene-transfer machinery for delivery inside the target host cell. The mechanism of ssRNA ejection from the capsid and injection into the host is poorly understood, due in part to the high level of secondary structure of the RNA that it is assigned to the type like a DNA plasmid transfer [64]. Qβ is classified as a male dependent virus, since it uses F-pili to initiate infection. As a (+) ssRNA, Qβ will serve as mRNA for early protein synthesis like the subunit of the polymerase complex (Rp) and a template for multiple genomic copy synthesis [65]. The life cycle is promoted by the secondary structure of the RNA [65]. Engineering some domains of this ssRNA has substantially modified the phage titer over the time. Excess phage progeny components can disturb the host functions or lyse it. The mechanism involved in the final step, following assembly of phage components into new progenies, is not clear for Qβ. Freely generated progenies would, in turn, start their infection cycle by adsorbing to the F-pili. Additionally, the Qβ cycle takes about 50 min at 37 °C and releases about 100 progenies [66,67,68].

#### 3.1.2. Proteins Involved in the Life Cycle and Functions

The A_2_ protein allows each phage particle to be adsorbed to the F-pili. The A_2_ protein is complexed with Qβ phage RNA, and is always cleaved to eject it from the particle. There is no distinguishable lysis protein (LP) gene in the Qβ genome. Recently, the A_2_ protein was found to carry out cell lysis through another pathway [69]. Thus, the A_2_ protein plays two critical functions in the phage cycle consisting of initiation and termination. The Rp is the β-subunit of the RNA-dependent-RNA-polymerase, (RdRp) together with the host elongation factors EF-Tu, EF-Ts, and the ribosomal protein S1 [70]. The Rp hijacked those host proteins and exploited them to replicate the phage’s genomic RNA. The function of the A_1_ is not entirely clear, but seems to play a role in the initiation of mature particles assembly. The Cp seems not to have a role in the infectivity process, other than to protect the phage RNA.

#### 3.1.3. Unknown or Unclear Part of the Cycle

The host penetration by Qβ RNA, as well as the liberation of the phage progenies, is unclear, but well adapted in many bacteria study thus far. The process of A_2_ cleavage and genome ejection is also not clear, but could be associated with the spontaneous retraction of the pilus. The mechanism of the β-subunit recruiting the host machinery for phage RNA replication is also not well reported, however, is adapted to the RNA replication system. The portion of the phage RNA recognized by the Rp is also not known.

### 3.2. Coliphage Qβ A_2_ Protein

#### 3.2.1. Structure and Domain

The structure of the A_2_ protein was recently resolved by Rumnieks and Tars [66,67,68]. Qβ contains only one copy of this protein, which is about 48.55 kDa. A_2_ is highly asymmetric in shape and spans 110 Å in length while bound to the phage genome. The A_2_ takes the position of a Cp-dimer and disrupts the perfect icosahedral symmetric shape of the capsid with the triangulation number of T = 3 [71,72,73]. The structural organization of the A_2_ has two distinct regions, namely an α-helical part (with four-helix core) and a β stranded part (with seven-stranded sheet). Additionally, at the tip of the A_2_ protein is a central portion with a five-stranded sheet [66,74].

#### 3.2.2. Functional Domains

The bond between the A_2_ protein and the phage RNA is through two positively charged areas on opposite sides of the α-helix. The A_2_-RNA complex provides protection to the genome against external ribonucleases and also initiates the formation of mature nascent virions in the infected host. The complex enters the packaged capsid where the A_2_ remains, partially exposed upon the phage surface. In this position, the rest of the A_2_ protein within the phage capsid binds to each of the surrounding protein coat dimers [74]. Although the interaction between A_2_ and the Cp dimers is very weak, together with the charge mediated interaction with RNA, it renders the mature A_2_ protein modestly soluble and difficult to purify. The position of the A_2_ allows for it to act as a minor structural constituent as the A_1_. The A_2_ drives phage tropism during infection by adsorbing the phage particles onto bacteria pili. The F-pili serves as a cellular receptor for the Qβ phage. In addition, the mature A_2_ mediates RNA ejection from the capsid and its penetration into the bacteria cytosol using the pilus canal.

The A_2_ protein was found to block the bacterial MurA enzyme of the murein precursor biosynthesis pathway, thereby promoting host cell lysis [75,76]. In the absence of a distinct lysis protein in Qβ, the A_2_ is suspected of playing the role of cell lysis. The mechanism of A_2_ mediated cell lysis is not clear. Nevertheless, it has been shown that overexpression of A_2_ might trigger cell lysis. The entire cDNA of the Qβ phage was cloned into several plasmids and used to produce phage without lysis of the host cell. Together, with the promising new advances in evolution and the uncover on the Qβ genome, we suggest that more factors than A_2_ mediating cell lysis are imminent [55,56,57,58,77,78,79,80].

#### 3.2.3. Contribution in Panning for Phage Display

Phage display libraries can be screened against several targets, including purified biomolecules (antibody, receptors), peptides binding to biological targets like cells or tissues, peptides binding to nonbiological targets like semiconductors and even environmental pollutant insecticides such as imidacloprid. Conventionally, peptides recognizing a target of interest are enriched from phage display libraries through an affinity selection-based procedure referred to as bio-panning. During this screening process, a population of recombinant phage particles is incubated with the target for a period of time, after which unbound targets are removed through a series of stringent washing steps. Different elution conditions are used to improve recombinant phage recovery from the immobilized target. Tight antibody-antigen (Ab-Ag) binding and attachment to solid support often requires heated, low pH-value buffer treatment [1,2,3,4,5,6,7,8,9,10] that frequently reduces the viable variants. We have succeeded in establishing an improved, optimized subtractive panning method to select and enrich antibody-specific antigens more efficiently, without any elution [55]. Our method of panning using the recombinant Qβ display platform, the A_2_ protein promotes infection while the phage variant is attached to the target, creating a unique and novel strategy for biopanning [55].

### 3.3. Coliphage Qβ Coat Protein

#### 3.3.1. Structure and Domain

The genome of Qβ is surrounded by a protective icosahedral shell that comprises 178 capsomers. Normally, the number of capsomers expected to make up a perfect symmetry is 180. However, there is a subtraction as a result of Cp dimer associated with the genomic RNA represented now by A_2_ protein [81]. Structurally, the capsomer is made up of 132 amino acids, that form a major core with a five-stranded anti parallel β-sheet, which is surrounded by a hairpin and two contiguous β-helices [82,83,84]. Disulfide bonds (S-S) link the capsomers into covalent pentamers and hexamers [84] in a stoichiometric ratio of 12:20, which is well-known in crystal structural studies of this major protein.

#### 3.3.2. Function of Domain

The major role of the Cp is to protect the RNA from any degradation and unfavorable environmental conditions. The Cp plays no other major role in the infection process. The presence of the phage particles on the pilus actively triggers the retraction necessary for RNA penetration and infection initiation. Additionally, the overexpression of Cp or capsids can induce cell lysis by bursting, as with any other viral infection system.

#### 3.3.3. Contribution to the RNA Display System

The Cp provides a platform for the exposition of a foreign peptide. The Cp is the major component of the platform and is therefore, the backbone of this RNA display system. The icosahedral shell of Qβ formed by the capsid polymer of the Cp, provides an equal distribution of the displayed peptides needed for genetic economy and avidity for immune related molecules.

### 3.4. Coliphage Qβ Read-Through or A_1_ Protein

#### 3.4.1. Structure and Domain

The crystal structure of the read-through domain of the A_1_ protein was determined at a 1.8 Å resolution [85]. The A_1_ protein represents a C-terminally extended version of capsid protein that contains 12 copies [55,86]. Structurally, the A_1_ is roughly globular in shape, with a mixed α/β architecture that is not observed in any other proteins [85]. The N-terminal part of the extension forms an unusually long polyproline type II helix [85,86,87]. In cryo-EM reconstructions of Qβ, there are no traces of the A_1_ extension, suggesting that copies of A_1_ might be flexibly attached and randomly distributed in individual phage particles. The read-through domain of the A_1_ protein is made up of five-stranded β-barrels, a β-hairpin, and a three-stranded β-sheet [85,86,87].

#### 3.4.2. Functional Domains

The A_1_ protein is required for infection, but the exact function of the A_1_ protein is unknown. The A_1_ protein is a read-through product of the coat gene stop codon [86]. It is expressed through incorrect reading of the leaky stop codon of the coat protein by the replicase [86]. The occasional read-through mechanism is a means of regulation and is an advanced step in phage evolution, which is very important for the evolutionary phage display system described here and is unique to this type of phages. The long stretch residues of polyproline type II helix are known to foster protein-protein and protein-nucleic acid interactions, which is crucial for mature phage formation [85,86,87].

#### 3.4.3. Contribution to the Qβ Phage Display Platform

The A_1_ protein shares the same initiation codon with the coat protein and is produced during the UGA stop codon triplet (about 400 nucleotides from the initiation) readthrough [86,87,88,89]. In addition, it is suppressed by a low level of translation, where the coat protein’s UGA stop codon triplet (about 400 nucleotides from the initiation) ribosome misincorporates tryptophan [86]. This suppression of the stop and extension of translation is the target of foreign gene insertion for phage display. We have successfully deleted up to 150 nucleotides at the C-terminal of the A_1_ gene without disrupting infectivity and shape of Qβ particles formed. So far, up to 300 nucleotides can been added at the C-terminal end of the A_1_ protein [55,56,57]. Thus, the A_1_ protein as the minor coat protein serves to present the inserted probe upon the surface of the recombinant phage, thereby playing a critical role not only in this novel technology but equally in the directed evolutionary phage display system.

### 3.5. Coliphage Qβ Replicase Protein

#### 3.5.1. Structure

The replicase protein of Qβ is an RNA dependent RNA polymerase (RdRp). The phage genome encodes only for the β-subunit of the RdRp replicase. Replicase is a holoenzyme made up of four subunits consisting of a 65 kDa β-subunit encoded by the phage and three others host encoded subunits, including the 70 kDa α ribosomal protein (S1), the 45 kDa elongation factor, γ (EF-Tu) and 35 kDa elongation factor δ (EF-Ts) [90]. The S1-γ subunit forms part of the bacterial 70 S ribosome and is one of 21 proteins that are involved in the translation of mRNA in the cell after transcription. The sedimentation ratio of the 70 kDa S1 protein is lower than anticipated, due to its elongated shape [91]. Overall, four antiparallel β-sheets and six α-helices form the finger domain. Then, two bundles of 3 α-helices and 3 antiparallel β-sheets form the thumb. In total, five antiparallel β-sheets flanked by four α-helices form the palm [92].

#### 3.5.2. Subunit Definition

The β-subunit is made up of 3 domains, including a finger, thumb, and palm. The two elongation factor subunits γ and δ (EF-Tu and EF-Ts) are bound tightly through hydrophobic interactions to both the finger and thumb domains [90,93]. Specificity is assured between the template and polymerase by the interaction between the virus and the host. The right-handed S1 protein structure is made up of a finger, thumb, and palm domain [70,94]. Translational elongation factor subunit γ binds to aminoacylated tRNAs (aatRNA) and subsequently moves them to the correct position on the ribosome A-site, by forming a ternary complex of EF-Tu: GTP: aatRNA [94]. Hydrolyzing GTP to GDP+ inorganic phosphate provides the energy to form this complex and for its release from the ribosome, thus leading to accurate translation [95]. The second elongation factor of *E. coli*, elongation factor subunit δ, is another factor allowing the hydrolsis of GTP to GDP. The release of the bound GDP by elongation factor subunit γ allows it to be recycled and to bind a new GTP, which then catalyzes another aatRNA.

When Qβ phage infects a bacterial host cell, the three subunits of the bacteria replicative enzymes are hijacked by the β-subunit thereby forming a replicase holoenzyme complex [96]. Here the S1 becomes the α subunit while the EF-Tu, and EF-Ts become the γ and δ subunits, respectively. The core complex of the replicase is therefore made up of the β subunit, EF-Tu and EF-Ts which in combination performs the function of polymerization [92,93,94,95,96]. There is also a 102 amino acid enzyme host factors (HF1) which is encoded by the bacteria *hfq* gene. This factor is required for the initiation of replication and must be present for replication to occur [97]. During the replication process, the ratio between HF1 and the RNA is the rate determining factor instead of the ratio between RNA and the replicase [97,98]. For replication of Qβ’s positive-stranded genome to begin, the 215 kDa replicase holoenzyme must be completely assembled from the S1 ribosomal protein, as well as the EF-Tu and EF-Ts elongation factors. Complementary negative stranded RNA is first synthesized, and this will be used as the template to synthesize more positive stranded RNA, eventually serving as mRNA. During replication process, both the positive and negative strands act as templates, implying that after each round of replication, there is doubling of the phage genome [15,16,96]. Qβ can use both positive and negative stranded RNA and replicase to amplify its genome 10^4^-fold in less than an hour. This signifies an exponential growth rate driven by a strong replicative strength.

#### 3.5.3. Contribution to Evolution and Phage Display

Qβ’s replicase is not like many other replicases, having some unique characteristics. Additionally, its β-subunit can select its own RNA template amongst a large amounts of other host RNAs. The replicase replicates only its own RNA by making it more than 10^4^ times in less than an hour. These two steps are also done without using endogenous primers [99,100]. S1 protein and the EF-Tu recognize the template. Strand recognition by EF-Tu: EF-Ts is through the CCC motif of the CCCA sequence which is located on the 3′ end of the RNA template [100]. Equally, this recognition is done on the recombinant RNA as well and is important in the replication of any vector used for this RNA phage display. The function of polymerization occurs once the 3′ bound end of the template enters the replicase initiation site [96,97,98]. Minus strands are not recognized by ribosomes, unlike the plus strands, and therefore must undergo a different mechanism of replication. The plus strand will replicate in equal amounts along the minus strand if HF1 is in excess. Replication is a linear process. Once the plus strand is copied by the replicase and the replication is terminated, the newly obtained minus strand then becomes a template through a switching or is a free-floating minus strand. At the free 3′ end, an A residue is added post transcriptionally [75,101]. The RdRp lacks the proofreading activity, which contributes greatly to the existence of Qβ phage as quasi-species [42,43,44,45,46,47,48,49,50,51,52,53]. The library variation is favored by the RdRp in the RNA phage display library. Certainly, the RdRp will be a key player in the adaptation of a displayed probe to its target during the process of amplification and novel panning with the Qβ phage display.

## 4. Coliphage Qβ Display System

### 4.1. Overall Methodology: Features and Emphasis

Although generating recombinant RNA (rRNA) vectors for phage display is very challenging, the RNA phage remains an ideal candidate for IVE. To overcome this challenge, our consortium has optimized a combination of three varieties of plasmids containing the reverse engineered full cDNA of the Qβ phage. All genetic engineering intending to generate vectors for phage RNA phage display is carried out on these plasmids. We have successfully created a multiple cloning site at the end of the A_1_ gene where unique restriction enzymes can be used for gene probe insertion. Traditionally, this multiple cloning site, present on the minor protein gene, is shared by all four proteins (p3, p6, p7, p9) of M13 phage. Additionally, these minor coat proteins exercise a combined vital role in the infection cycle of M13 and in display technology. The facts that protein (pIII) plays a role in the display technology by exposing the probes and is implicated in the initiation of infection cycle of the M13 phage are major drawbacks of the traditional M13 system. The multifunctionality of pIII compromises the platform, especially when used in displaying a library of peptides. This compels the use of acidic buffers for elution and neutralizing during biopanning. These harsh conditions are detrimental, both to infectivity and the affinity of phage variants arising from library. In contrast, the A_2_ protein of Qβ is involved in initiating the phage life cycle. In the RNA Qβ phage system, the two major functions of infection initiation and bioprobe display played by the pIII in the M13 phage display system are split between the A_1_ and A_2_ proteins. The sharing of these functions between A_1_ and A_2_, increases the specificity of Qβ phage display biotechnology and improves both the affinity and infectivity of phage variants during Qβ phage library production.

Being the most attractive RdRp, the Qβ replicase with its well-known promiscuity, is the key element of this RNA Qβ phage display system because it is responsible for driving diversity and inducing the adaptability of the variant populations to their corresponding target(s). These minor protein (A_1_ and A_2_) mediated features during Qβ phage display are unique and novel. The uniqueness of the RNA coliphage Qβ display system renders it a powerful tool in IVE of peptides, antibodies, and nanobodies.

Another feature of the Qβ phage display system is the icosahedral structure of variants and the equidistance position of the fusion protein A_1_-probe(s) on the surface of the phage which is an attractive tool for affinity or avidity study for appropriate target. This A_1_-probe arrangement eliminates the need for harsh acidic elutions during the recombinat phages screening process. The novel biopanning strategy developed with Qβ is quick, permitting more than 10 possible rounds per day which, significantly, facilitates the implementation of RNA phage display in synthetic biology. During this novel biopanning strategy, the elution of variants is done by infection, which avoids the harsh conditions associated with acidic treatment and neutralization on this major step in the conventional panning.

### 4.2. Determining Steps in the RNA Qβ Display System

As a new tool in IVE, a critical pre-requisite is the maximum peptide(s) size potentially acceptable upon the exterior surface of the recombinant Qβ phage particle without compromising its infectivity. Another essential necessity would be the relation between peptide motives and the ability of the recombinant Qβ phage to expose them. Through sequential deletions in the A_1_ gene, we have succeeded to create more room for the insertion of foreign DNA. However, the effect of such deletions (gene size reduction) in A_1_ must be ascertained to further improve the platform. Given the variation observed in phage host cell expression, where different host cells are tested, including *E. coli* K12, Q13, Hfrh, and HB101, A_2_ protein’s involvement in host cell lysis cannot be the only means through which Qβ mediates host cell lysis. Knowing the mechanism of Qβ mediated host lysis during infection would help to improve the production and the effective use of the Qβ display platform. The high rate of mutation within this phage display platform could be an indication of the extent of evolution, which must eventually be determined for the system. Addressing these listed knowledge gaps within the coliphage Qβ display system will further augment its suitability and uniqueness for directed evolutionary biotechnology and IVE investigations.

## 5. Application in Directed Evolution and Conclusion

### Application

**Figure 2:**
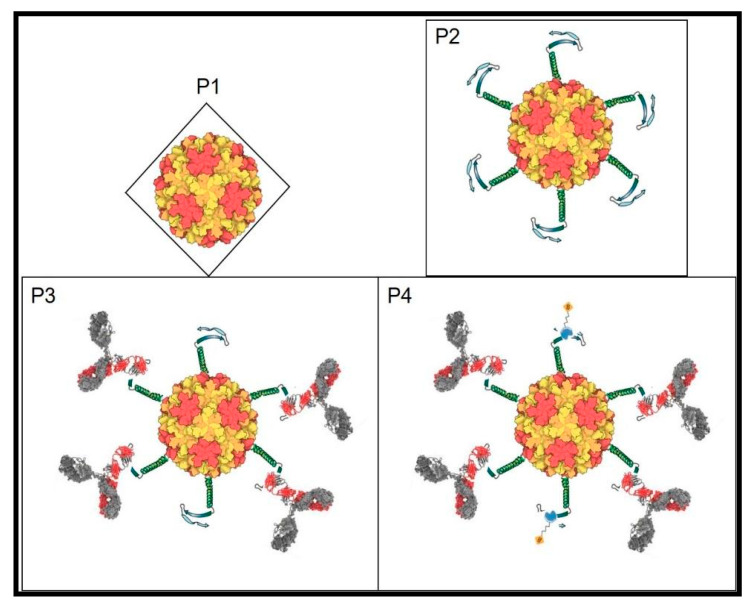
RNA coliphage Qβ structure with T = 3; P1: Qβ phage icosahedral shell; P2: Qβ phage (P1) displaying a probe and a transducer peptides joined by a linker all fused to the end of the A_1_ protein; P3: RNA Qβ phage exposing the probe (here an RNA virus epitope) complexing its corresponding IgG through the variable domain and hiding the transducer; P4: P3 detection of the remaining transducer by binding to the analyte in real time and quantifiable. Without probe-target (here antibody-antigen) complex, the detection is 100% measurable (transducer saturated with detector) and the opposite (antibodies compete and hide the transducer) will be subtracted to quantify the target detected by the biosensor.

We have reviewed the genome and proteins of the coliphage Qβ and proposed ways to improve its potential to display peptides, antibodies, nanobodies, and different library formats through genetic engineering. In contrast to the traditional display with DNA phages, this new system has multiple advantages ranging from the rRNA construction, phages production, to biopanning for variants selection. This has broad application in virology especially through displaying epitopes (mimotopes) of challenging RNA viruses which would help in the development of both vaccines and optimize novel biomarkers for point of care kits or theragnosis. In cancer biology, random library display can lead to the selection of major immune checkpoint inhibitors for therapeutic purposes. Generalized library screens can aid in developing peptides as new drugs from library selection and evolution. The introduction of new biomolecules resulting from mixtures of selected peptides from novel libraries and appropriate nanoparticles becomes feasible. Similarly, construction of novel biosensors by evolving peptides to detect molecules and life-threatening agents can be developed as well (See Figure 2 attached) in the environment. The uniqueness of the RNA coliphage Qβ display system comes from its inherent structure, biology, and its potential broad application in live sciences.

## 6. Conclusions

The RNA coliphage display concept and methodology presented here, though in its infancy, represents a novel breakthrough strategyin phage display biotechnology that will strengthen molecular evolution, thereby expediting biomolecule identification and valorization. The major biotechnological applications stem from the direct evolution of these biomolecules on targets. The RNA phage expressing the probe binds to the target, while being allowed to infect the host cell for genomic replication and expression, thus leading to the production of probe progenies. Any pressure on the target-probe connection is obvious seocndary to new progenies, translating into direct evolution. For the first time, binding of the probe (phenotype) to its target, in connection with external pressure on the gene library (genotype), and continuous replication (directed evolution) combines to make RNA coliphage Qβ to be both unique and feasible. Additionally, this tool will help mimic, measure, and demonstrate live evolutionary concepts, which can result in developing novel evolved biologics crucial for new drug discovery against challenging diseases.
